# Analysis of the factors that influence caregiver burden in preschool children with neurodevelopmental disorders

**DOI:** 10.1192/j.eurpsy.2026.11132

**Published:** 2026-07-31

**Authors:** S. Pérez-Sánchez, C. Pérez-Sánchez, E. Gonzalez-Reigadas

**Affiliations:** 1 Psychiatry; 2 Medicina Familia, Servicio Murciano de Salud, Murcia, Spain

## Abstract

**Introduction:**

Severe mental illness in early childhood not only has a significant impact on the patient but also on other contexts such as the family. Caregivers take on almost the entirety of care responsibilities with limited social and educational support. This responsibility exposes caregivers to intense burden with negative consequences.

**Objectives:**

To evaluate and quantify the burden of the primary caregiver in parents of children with neurodevelopmental disorders.

**Methods:**

A prospective study was designed using structured interviews with caregivers of patients between 6 months and 5 years of age with neurodevelopmental disorders who attended mental health services for evaluation or follow-up. The Zarit Burden Interview was used for quantitative assessment. Diagnosis, primary caregiver, and social-health resources were recorded.

**Results:**

Out of a total of 35 patient visits with NDD between 11 months and 5 years of age, caregiver burden was observed to be severe in 42.2% of cases, and moderate in 21%. The primary caregiver was the mother. 27.5% made adequate use of social-health resources, while 42.7% had not requested them. The most frequently identified needs were financial and rehabilitative.

**Conclusions:**

Neurodevelopmental disorders present since early childhood lead families to significant destabilization of the family unit and psychiatric comorbidities in caregivers. Proper guidance and individual assessment of cases are necessary in order to inform families about the resources available in the social-health network, thereby preventing high caregiver burden and improving the quality of life of families.

**Disclosure of Interest:**

None Declared

